# Economic Optimized Medium for Tensio-Active Agent Production by *Candida sphaerica* UCP0995 and Application in the Removal of Hydrophobic Contaminant from Sand

**DOI:** 10.3390/ijms12042463

**Published:** 2011-04-08

**Authors:** Juliana M. Luna, Raquel D. Rufino, Clarissa D.C. Albuquerque, Leonie A. Sarubbo, Galba M. Campos-Takaki

**Affiliations:** 1 Post-Graduate Program in Biological Sciences, Federal University of Pernambuco, CEP 50.670–420, Recife, PE, Brazil; E-Mail: juliana_mouraluna@yahoo.com.br; 2 Nucleus for Research in Environmental in Environmental Sciences, Catholic University of Pernambuco, Rua Nunes Machado, 42, Bloco J, Térreo, Boa Vista, CEP 50.050–590, Recife, PE, Brazil; E-Mails: raqueldrufino@yahoo.com.br (R.D.R.); albqqs@yahoo.com.br (C.D.C.A.); leonie@unicap.br (L.A.S.); 3 Post-Graduate Program in Fungal Biology, Federal University of Pernambuco, CEP 50.670–420, Recife, PE, Brazil; 4 Center for Sciences and Technology, Catholic University of Pernambuco, Rua do Príncipe, Boa Vista, CEP 50.050–900, Recife, PE, Brazil

**Keywords:** factorial design, central composite design, optimization, *Candida sphaerica*, tensio-active agent

## Abstract

Statistical experimental designs and response surface methodology were employed to optimize the concentrations of agroindustrial residues as soybean oil (SORR) from refinery, and corn steep liquor (CSL) from corn industry, for tensio-active agent produced by *Candida sphaerica* UCP 0995. Three 2^2^ full factorial design were applied sequentially to investigate the effects of the concentrations and interactions of soybean oil refinery residue and corn steep liquor on the surface tension of free-cell culture broth for 144 h. Two 2^2^ central composite designs and response surface methodology were adopted to derive a statistical model to measure the effect of SORR and CSL on the surface tension of the free-cell culture broth for 144 h. The regression equation obtained from the experimental data using a central composite design was solved, and by analyzing the response surface contour plots, the optimal concentrations of the constituents of the medium were determined: 8.63% v/v (≅9% v/v) of SORR and 8.80% v/v (≅9% v/v) CSL. The minimum surface tension predicted and experimentally confirmed was 25.25 mN/m. The new biosurfactant, denominated Lunasan, recovered 95% of motor oil adsorbed in a sand sample, thus showing great potential for use in bioremediation processes, especially in the petroleum industry.

## 1. Introduction

Surfactants are surface active agents with wide ranging properties which include the lowering of the surface and interfacial tensions of liquids. Surface tension is defined as the free surface enthalpy per unit area and is the force acting on the surface of a liquid leading to minimization of the area of that surface. There are both synthetic and natural surfactants that are capable of reducing the surface tension of water from 72 mN/m to around 27 mN/m [1].

Optimizing the composition of the medium is an important issue in developing economically feasible biosurfactant production processes. Microbially produced surfactants or biosurfactants have attracted attention because of their low toxicity, biodegradability, and ecological acceptability. However, they must compete with surfactants of petrochemical origin in three respects—cost, functionality, and production capacity to meet the needs of the intended application [2].

Reducing the overall costs of biosurfactant production usually depends on improving the strain, the use of low cost raw materials such as agricultural and industrial wastes as substrates, the use of process scale-up and the use of advanced computer—based techniques for process control and optimization [3]. Carbohydrates and vegetable oils are among the most widely used substrates for research on biosurfactant production. Selection of waste substrates involves the difficulty of finding a waste with the right balance of carbohydrates and lipids to support optimal growth and production. Agroindustrial wastes with high carbohydrate or lipid content and urban wastes meet the requirements for use as substrates for biosurfactant production [4]. Soybean oil refinery residue (SORR) and corn steep liquor (CSL) are two examples of inexpensive agroindustrial wastes residues, rich in nutrients, which can be used for the production of biosurfactant so as to reduce process costs.

However, reports have not been found on optimizing SORR and CSL as medium components for biosurfactant production by *Candida sphaerica* UCP 0995. Therefore, the aim of this study was to optimize low cost medium based on soybean oil refinery residue and corn steep liquor for biosurfactant production by *C. sphaerica* UCP 0995 using full factorial designs (FFD) and a response surface methodology (RSM). Surface tension, the physical property conventionally used to detect biosurfactant production, was used as the response variable. The traditional one-factor at a time approach to optimization is time-consuming and incapable of reaching the true optimum due especially to its interaction with other factors [5]. The factorial design of optimization experiments is especially suitable for taking interactions into account and it is efficient. A combination of factors that generate a certain optimum response can be identified through factorial design and the use of response surface methodology [6]. Optimization through factorial design and RSM is a common practice in biotechnology for the optimization of media components and culture conditions [7–8]. This method has also been successfully applied in the optimization of a medium for biosurfactant production [9–10]. The aim of this investigation was to optimize the tenso-active agent by *Candida sphaerica* using agroindustrial residues, such as carbon (SORR) and nitrogen (CSL) sources, and its application in order to remove motor oil that is polluting sand.

## 2. Results and Discussion

This paper describes the use of SORR and CSL as low cost medium components for biosurfactant production by *C. sphaerica* UCP 0995. This production was detected by surface tension becoming lower when the microorganism was cultivated on water to which SORR and CSL were added, in various concentrations. The optimization process was conducted by using three two level 2^2^ full factorial designs followed by a two-level two factor central composite design. All experiments were performed in duplicate and the average of surface tension was considered as response.

### 2.1. Screening Experiments

In order to find the optimal concentrations of SORR and CSL for biosurfactant production, initially, a set of three two-level full factorial designs were conducted to investigate the effects and interactions of SORR and CSL on surface tension. The definitions of the factors and levels used in the factorial designs are presented in Table 1. The experimental designs carried out and results obtained are given in Table 2 and Table 3 (introduce later), respectively. The steepest ascent was used to determine the direction toward lower surface tensions. After the second step on the path, further experimentation did not manage to decrease surface tension further.

### 2.2. First Full Factorial Design

**Figure 1.**
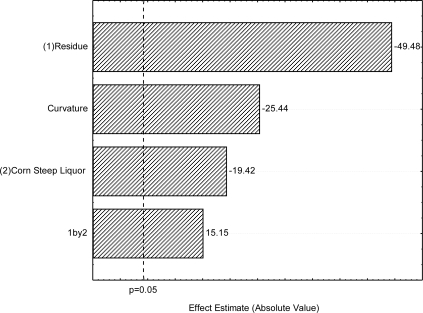
First 2^2^ full factorial design—Pareto’s Chart of standardized effects for **(1)** soybean oil refinery residue and **(2)** corn steep liquor using surface tension as response variable. The point at which the effect estimates were statistically significant (p = 0.050) is indicated by dashed line.

The effects of SORR and CSL on surface tension, as well as the interaction between them, in the first 2^2^ full factorial designs are shown in Figure 1. The Pareto chart clearly shows that SORR concentration is by far the most important factor affecting the reduction of surface tension of cell-free culture broth, followed by CSL concentration and SORR-CSL interaction. As can be seen in the Pareto chart, the increase in SORR concentration from 0 to 6% (v/v) influenced negatively, in a statistically significant way, the increase in surface tension, leading to lower surface tension. The increase in CSL concentration from to 6 % (v/v) also influenced negatively, in a statistically significant way, the increase in surface tension, leading to lower surface tension. On the other hand, the SORR-CSL interaction contributes in a statistically significant way to the increase of surface tension in the culture medium. Curvature check [11] was carried out and revealed lack of fit of the linear approximation. Figure 1 also shows that curvature effect crosses the 95% confidence level, indicating the proximity of the optimum point.

### 2.3. Second Full Factorial Design

**Figure 2.**
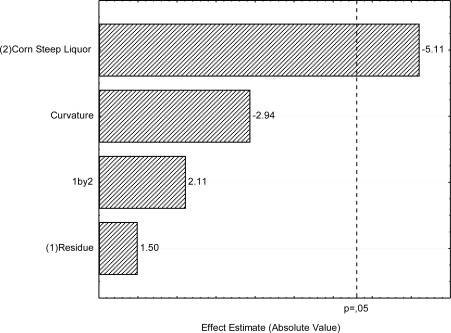
Second 2^2^ full factorial design—Pareto’s Chart of standardized effects for **(1)** soybean oil refinery residue and **(2)** corn steep liquor using surface tension as response variable. The point at which the effect estimates were statistically significant (p = 0.050) is indicated by dashed line.

Given the results of the first design, the SORR and CSL concentrations were increased from 6 to 12% (v/v), as per Table 1. The effects of SORR and CSL concentrations on surface tension, as well as the interaction between them, in the second 2^2^ full factorial designs are shown in Table 2 and Figure 2. The interpretation of the factorial design by Pareto chart in Figure 2, using surface tension as response, shows that the only significant variable for surface tension was the CSL concentration. The increase in the CSL concentration from 6 to 12% (v/v) influenced negatively, in a statistically significant way, the increase in surface tension, leading to lower surface tension. On the other hand, the increase of the SORR concentration, the curvature effect and the SORR-CSL interaction were not statistically significant. Although the curvature check was carried out and revealed lack of fit of the linear approximation, in this design the curvature effect was not statistically significant.

### 2.4. Third Full Factorial Design

**Figure 3.**
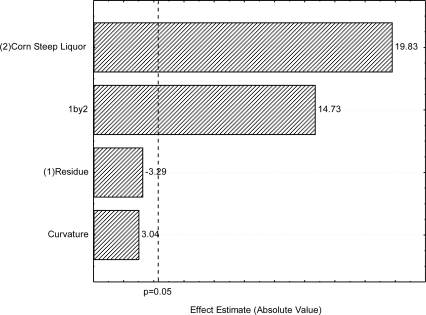
Third 2^2^ full factorial design—Pareto’s Chart of standardized effects for **(1)** soybean oil refinery residue and **(2)** corn steep liquor using surface tension as response variable. The point at which the effect estimates were statistically significant (p = 0.050) is indicated by dashed line.

Given the results of the second design, the CSL concentration was increased from 9 to 12% (v/v) (Table 3), as per Table 1. However, the levels of SORR were not modified. As can be seen in the Pareto chart (Figure 3), the increase of the CSL concentration from 9 to 15% (v/v) and that of the SORR-CSL interaction produced statistically significant positive effects on the increase in surface tension, leading to higher surface tension. On the other hand, in the conditions studied, the CSL concentration produced a negative but not statistically significant effect on the increase in surface tension. Although the curvature check was carried out and revealed lack of fit of the linear approximation, in this design the curvature effect was not statistically significant either.

### 2.5. Optimization Experiment

The strategy used to attain the goal of this study was to explore the experimental space around the selected conditions by analyzing the results of the three full factorial designs carried out. After the third full factorial design, further experimentation does not manage to decrease the surface tension of the cell-free broth. Such results indicated that the SORR and CSL concentrations of the second design were near optimal. Thus a central composite design was carried out to achieve the lowest surface tension of the cell-free broth and optimize the medium composition. The factor levels used for the 2^2^ composite central designs (Table 2) were chosen based on the results of the previous 2^2^ full factorial designs (Table 1). The results for the central composite design are presented in Table 4. The experiments showed that low surface tension of the cell-free broth was obtained under all test conditions studied and that the lowest surface tensions of the cell-free broth were found at central level conditions (zero level—runs numbers 9, 10 and 11). The average surface tension of the cell-free broth at zero level was 25.29 ± 0.21 mN/m.

The results obtained from the central composite design were fitted to a second order polynomial equation to explain the dependence of surface tension on the components of the medium.

(1) $$\text{z}=25.284-0.288\text{x}+0.718{\text{x}}^{2}+0.004\text{y}+0.374{\text{y}}^{2}+0.214\text{xy}$$

where *z* is the predicted response of surface tension, *x* and *y* are the coded values of SORR and CSL respectively.

**Figure 4.**
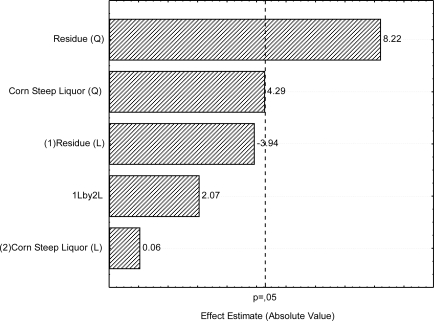
Central composite design—Pareto’s Chart of standardized effects for **(1)** soybean oil refinery residue and **(2)** corn steep liquor using surface tension as response variable. The point at which the effect estimates were statistically significant (p = 0.050) is indicated by dashed line.

The model allowed the evaluation of the effects of linear, quadratic and combined effects of the independent variables upon the dependent variable. The significance of each coefficient of the regression equation was determined by the Student *t*-test and *p*-values, which are graphically represented in the Pareto chart illustrated in Figure 4. Analysis of the results in Figure 4 shows that the SOOR concentration was variable and had the greatest influence on the surface tension of the free-cell broth. The quadratic contribution of SORR was positive and significant. The linear contribution of SORR was also negative but not statistically significant. The quadratic contribution of the CSL was positive and very near to being of statistical significance. The linear contribution of the CSL and the SORR_CSL interaction were positive but not statistically significant. The results showed that the concentrations of the SORR and CSL should be maintained close to the center point to obtain the maximum response.

The analysis of variance (ANOVA) of the second order model shows that the model is significant (Table 5), as is evident from the Fisher F test, where the calculated F value (F_model_ = 15.51) is greater than the tabular F value (F_tab_ = 5.05). The value of the determination coefficient (R^2^ = 0.87) indicates that 87% of the variability in the response could be explained by the second-order polynomial prediction equation, as expressed in Equation (1), demonstrating the goodness of fit of the model. The value of the adjusted determination coefficient (Adj. R^2^ = 0.73)—also indicates the significance of the model. A good correlation between the experimental and predicted values was obtained once the correlation coefficient (R = 0.87) is close to 1. The model did not show lack-of-fit (lof), as is evident from the Fisher F test, where the calculated F value (F_lof_ = 4.08) is not greater than the tabular F value (F_tab_ = 19.2).

**Figure 5.**
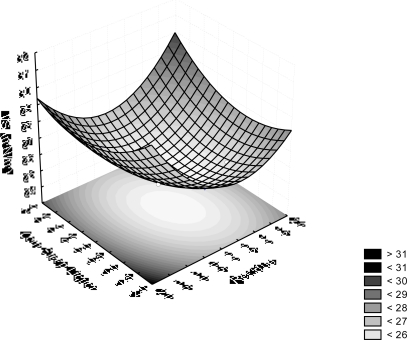
Surface tension response surface showing interaction between soybean oil refinery residue and corn steep liquor concentrations.

By means of Equation (1), the optimum coded values for SORR and CSL concentrations, were found to be respectively 0.21 and −0.07. The corresponding optimum uncoded values were calculated to be 8.63% (v/v) and 8.80% (v/v), respectively. The minimum surface tension of the free-cell broth predicted in these optimum conditions was 25.25 mN/m. These results are shown graphically in Figure 5, through the three-dimensional plot of SORR and CSL concentrations against surface tension of the cell-free broth.

The model was validated by repeating the experiments under the optimized conditions, which resulted in a surface tension of 25.34 ± 0.16 mN/m, thus proving the validity of the model. For simplicity’s sake, the SORR and CSL concentrations were rounded to 9% (v/v). New experiments were performed to verify the predicted optimum. The result (25.53 ± 0.24) from three replications (25.76, 25.5 and 25.29% v/v) was coincident with the predicted value and once again the model was proven to be adequate.

The strategy of optimization using sequential factorial design was also employed in our laboratories to enhance the tension-active emulsifying agent produced by *C. lipolytica* using soybean oil refinery residue as substrate. A full factorial design was used to evaluate the effect of residue, glutamic acid and yeast extract concentrations. It was found that the increase of the residue concentration led to a decrease in the surface tension, and more so if the glutamic acid concentration was raised at the same time. The medium surface tension reached 25.29 mN/m [12].

In another study, the biosurfactant from *C. sphaerica* cultivated in a low cost medium formulated with 5% soybean refinery residue and 2.5% corn steep liquor as substrates reduced the surface tension to values around 26 mN/m after 144 h [13], while the biosurfactant from *C. lipolytica* cultivated in a mineral medium supplemented with the same refinery residue used in this study reduced the surface tension to 32 mN/m [14].

Suitable models have been established to describe the response of the experiments to biosurfactant production by probiotic bacteria. The replacement of the synthetic media by cheaper alternative media, such as cheese whey and molasses led to an increase of about 1.2–1.5 times in the mass of produced biosurfactant and a 60–80% reduction in the preparation costs of the medium [13].

Statistical optimization of medium components for the production of bioemulsifier by *Candida lipolytica* in sea water was performed using experimental design and surface response methodologies. A mathematical model to represent the emulsification activity, as a function of urea, ammonium sulfate e potassium dihydrogen orthophosphate concentrations, was proposed. The model did not show lack-of-fit and the determination coefficient indicated that the model could explain 95.72% of the variability in the emulsification activity. According to the model, the urea, ammonium sulfate and potassium dihydrogen orthophosphate concentrations necessary to attain the maximum emulsification activity (4.415 UEA) were respectively 0.544 % (w/v), 2.131 % (w/v) and 2.628 % (w/v). Validation of the model was accomplished by experiments carried out on optimized medium conditions [10,15].

Experimental design tools were used to study the effects of process conditions on surfactant production during batch tests conducted using a strain of *Pseudomonas alcaligenes* growing on palm oil as the sole carbon source. A variable screening procedure showed that the surfactant production depended primarily on the palm oil concentration, with a decrease in surface tension from 54 to 31 mN/m at 48 h of the bioprocess [16].

Important parameters for biosurfactant production from *Serratia* sp. SVGG16 were selected through experimental design. Results demonstrated that this strain was able to reduce surface tension of the medium to 34 mN/m [17].

### 2.6. Application of the Biosurfactant in the Removal of Hydrophobic Contaminant Adsorbed in Sand

Biosurfactants can emulsify hydrocarbons enhancing their water solubility, decreasing surface tension and increasing the displacement of oil substances from soil particles [18].

The results obtained for the removal of motor oil adsorbed in the samples of sand tested by the cell-free culture medium from *C. sphaerica* obtained after 144 hours and by distilled water (control) showed the removal of 95 and 10% of the oil, respectively. Since the tests were conducted with the containing biosurfactant cell-free broth (crude biosurfactant), the results were considered satisfactory.

Promising results have been obtained by the biosurfactants produced from *Candida* species. Batista *et al.* [19] for the cell-free broth containing a biosurfactant produced by *C. tropicallis*, showed the recovery of 80% of residual crude oil adsorbed in the sand and Coimbra *et al.* [20] for the biosurfactants produced by *C. guilliermondii* and *C. lipolytica* showed the high ability of these molecules to remove motor oil and petroleum adsorbed in the samples of sand tested. Results described in the literature show that the biosurfactant from *C. antarctica* remove about 50% of the oil adsorbed in sand [21], while the biosurfactant produced by *C. sphaerica* cultivated in low-cost medium removes 65% of the motor oil adsorbed in beach sand [18]. More recently, the biosurfactant isolated from *C. glabrata* removed 84% of the motor oil [22].

## 3. Experimental Section

### 3.1. Microorganism

*Candida sphaerica* UCP 0995 was obtained from the culture collection of the Catholic University of Pernambuco, Brazil. The microorganism was maintained at 5 °C on Yeast Mold Agar (YMA) slants containing (w/v): yeast extract (0.3%), malt extract (0.3%), tryptone (0.5%), D-glucose (1.0%) and agar (5.0%). Transfers were made to fresh agar slants each month to maintain viability.

### 3.2. Agroindustrial Wastes

The SORR was used as the main carbon source while the CSL constituted the nitrogen source. Both agroindustrial byproducts also provided the other nutrients essential for the metabolism of yeast. The composition of the refinery residue was previously described by Rufino *et al.* [23]. Basically, CSL has 21–45% proteins, 20–26% lactic acid, 8% ashes (containing Ca^2+^, Mg^2+^, K^+^), 3% sugars and a low fat content (0.9–1.2%).

### 3.3. Biosurfactant Production

All optimization experiments were carried out in 250mL Erlenmeyer flasks containing 100 mL of basal medium. The basal medium consisted of SORR and CSL dissolved in distilled water, in various concentrations, according to the experimental designs. The media were sterilized by autoclaving at 121 °C for 20 min. The pH of the media was adjusted to 5.3. The inoculum of *C. sphaerica* UCP 0995 was prepared by transferring cells grown on a slant to 50 mL of Yeast Mold Broth (YMB). The seed culture was incubated for 24 h at 28 °C and agitated at 150 rpm. Each flask (250 mL) containing basal medium was inoculated with 1% (v/v) seed culture (10^4^ cells/mL). The flasks were incubated on a rotatory shaker at 150 rpm, for 144 h at 27 °C. At regular intervals, samples were withdrawn for analyses. All the assays were carried out in duplicate.

### 3.4. Surface Activity Assay

Surface tension was determined on cell-free broth obtained by centrifuging the cultures at 10,000 × g for 15 min with a Tensiometer model Sigma 70 (KSV Instruments Ltd., Finland) using the Du Nouy ring method at room temperature (±28 °C). Measurements of surface tension from distilled water and from the mineral medium were used as controls [24].

### 3.5. Optimization of the Biosurfactant Production Medium

Sequential strategy based on the application of statistical experimental design associated with RSM was used to optimize the biosurfactant production by *C. sphaerica* UCP 0995 in shake flask cultures. The choice of inexpensive raw materials is important to the overall economics of the biosurfactant production process because they account for 50% of the final product cost [25]. For this reason, SORR (carbohydrate-rich) and CSL (nitrogen-rich) were chosen as medium components, the concentrations of which should be optimized.

The production medium optimization process was carried out using three 2^2^ FFD and two 2^2^ CCD. The FFD were carried out to verify the effects and interactions of the SORR and CSL on the surface tension of the cell-free culture broth and the two CCD factors were applied to determine the optimal concentrations of soybean oil refinery residue and corn steep liquor, using the range and levels shown in Tables 1 and 2. The lowest and the highest levels were coded as −1 and +1, and the middle point as 0 (zero). The relation between the coded values and actual values were described as in the following equation:

(2) $$X_{i}=\frac{x_{i}-x_{0}}{\Delta x_{i}}$$

where *X_i_* is the coded value of the independent variable, *x_i_* is the actual value of the independent variable, *x_0_* is the actual value of the independent variable on the center point and Δ*x_i_* is the step change value. In all designs, triplicates at the center point were carried out to provide an estimate of pure error. The center point triplicates were also used to check for curvature in 2^2^ full factorial designs. The optimum values of the concentrations of SORR and CSL were obtained by solving the regression equation and also by analyzing the contour plots of the response surface [11–26]. To determine the significance of effects, an analysis of variance (ANOVA) with 95% confidence limits was used. The effects and significance of the variables were graphically illustrated using Pareto charts. A Pareto chart consists of bars with a length proportional to the absolute value of the estimated effects, divided by the standard error. When using the Pareto chart to analyze the effect of variance, estimates are sorted from the highest to the lowest absolute value. The chart includes a vertical line at the critical t-value for an alpha of 0.05. Effects for which the bars are smaller than the critical t-value are considered not to be significant, nor to affect the response variables. Effects may be positive or negative. Variance analysis, the determination of regression coefficients and graphs were conducted using Statistica® software version 6.0 (Statsoft.Inc, USA).

### 3.6. Application of the Biosurfactant in Removing Motor Oil from Contaminated Sand

Testing the suitability of biosurfactant for enhanced oil recovery was conducted by using 60.0 g of beach sand impregnated with 5.0 mL of motor oil. Biosurfactant produced by *C. sphaerica* cultivated in the optimized medium, comprising distilled water supplemented with 9% refinery residue plus 9% corn steep liquor, was used in the removal tests. Fractions of 20.0 g of the contaminated sand were transferred to 250 mL Erlenmeyer flasks, which were submitted to the following treatments: addition of 40 mL of the cell-free metabolic broth and addition of 40.0 mL distilled water (control). The samples were incubated on a rotary shaker (150 rpm) for 24 h at 27 °C and then were centrifuged at 5000 g for 10 min for separation of the wash solution and the sand. The amount of oil residing in the sand after the impact of biosurfactant was gravimetrically determined as the amount of material extracted from the sand by hexane [22].

## 4. Conclusions

The association of statistical design of experiments and surface response methodology proved to be effective for optimizing the medium for biosurfactant production. The results of the three 2^2^ experimental designs, and the two factor central composite design carried out, showed the ability of *Candida sphaerica* to grow in low cost medium consisting of SORR and CSL. The optimization of the medium using low cost constituents (8.63 ≅ 9.0% v/v of SOOR and 8.80 ≅ 9.0% v/v) resulted not only in reducing the surface tension of cell-free broth to 25.34 ± 0.16 mN/m, but also in reducing the cost of the medium. The possible application of the biosurfactant produced in the optimized medium in bioremediation processes of the petroleum industry was demonstrated by its ability to remove a hydrophobic contaminant.

## Figures and Tables

**Table 1.** Levels of the three 2^2^ full factorial designs.

	**First Full Factorial Design**		
**Factor**	**Level**		
	−1	0	+1
Soybean oil refinery residue (%v/v)	0	3	6
Corn steep liquor (%v/v)	0	3	6
	**Second Full Factorial Design**		
**Factor**	**Level**		
	−1	0	+1
Soybean oil refinery residue (%v/v)	6	9	12
Corn steep liquor (%v/v)	6	9	12
	**Third Full Factorial Design**		
**Factor**	**Level**		
	−1	0	+1
Soybean oil refinery residue (%v/v)	6	9	12
Corn steep liquor (%v/v)	9	12	15

**Table 2.** Levels of the two factor central composite design.

**Central Composite Design**					
**Factor**	**Level**				
	**−1.41**	**−1**	**0**	**+1**	**−1.41**
**Soybean oil residue (%v/v)**	3.68	5	8	11	12.32
**Corn steep liquor (%v/v)**	4.68	6	9	12	13.32

**Table 3.** Coded levels and surface tension results for the three 2^2^ full factorial designs.

**Assay**	**Soybean Oil Residue**	**Corn Steep Liquor**	**Surface Tension (mN/m)**		
			**First FFD**	**Second FFD**	**Third FFD**
1	−1	−1	68.64	25.50	25.17
2	1	−1	27.92	25.46	24.15
3	−1	1	46.86	25.09	25.46
4	1	1	25.23	25.29	26.11
5	0	0	30.55	25.25	25.29
6	0	0	29.92	25.23	25.37
7	0	0	29.29	25.14	25.40

**Table 4.** Coded levels and surface tension results for the two factor central composite design.

**Assay**	**Soybean Oil Residue**	**Corn Steep Liquor**	**Surface Tension (mN/m)**
1	−1	−1	26.71
2	1	−1	26.09
3	−1	1	26.50
4	1	1	26.73
5	−1.41	0	27.25
6	1.41	0	25.90
7	0	−1.41	26.03
8	0	1.41	25.75
9	0	0	25.50
10	0	0	25.09
11	0	0	25.25

**Table 5.** ANOVA results for the two factor composite central design.

**Source of variation**	**Sum of squares**	**Degrees of freedom**	**Mean square**	**F**
**Regression**	3.90	5	0.78	15.51
**Residual**	0.61	5	0.12	
**Lack of fit**	0.52	3	0.17	4.08
**Pure error**	0.09	2	0.05	
**Total**	4.51	10	0.45	

R^2^ = 0.87	R^2^_adj_ = 0.73	F_(0.95, 5 , 5)_ = 5.05	F_(0.95, 3 ,2)_ = 19.20	
